# Artificial intelligence–assisted phenotype discovery of fragile X syndrome in a population-based sample

**DOI:** 10.1038/s41436-021-01144-7

**Published:** 2021-03-26

**Authors:** Arezoo Movaghar, David Page, Danielle Scholze, Jinkuk Hong, Leann Smith DaWalt, Finn Kuusisto, Ron Stewart, Murray Brilliant, Marsha Mailick

**Affiliations:** 1 Waisman Center, University of Wisconsin–Madison, Madison, WI USA; 2 Department of Biostatistics and Bioinformatics, Duke University, Durham, NC USA; 3 Department of Pediatrics, University of Wisconsin School of Medicine and Public Health, Madison, WI USA; 4 Morgridge Institute for Research, Madison, WI USA; 5 Marshfield Clinic Research Institute, Marshfield, WI USA

## Abstract

**Purpose:**

Fragile X syndrome (FXS), the most prevalent inherited cause of intellectual disability, remains underdiagnosed in the general population. Clinical studies have shown that individuals with FXS have a complex health profile leading to unique clinical needs. However, the full impact of this X-linked disorder on the health of affected individuals is unclear and the prevalence of co-occurring conditions is unknown.

**Methods:**

We mined the longitudinal electronic health records from more than one million individuals to investigate the health characteristics of patients who have been clinically diagnosed with FXS. Additionally, using machine-learning approaches, we created predictive models to identify individuals with FXS in the general population.

**Results:**

Our discovery-oriented approach identified the associations of FXS with a wide range of medical conditions including circulatory, endocrine, digestive, and genitourinary, in addition to mental and neurological disorders. We successfully created predictive models to identify cases *five years prior to clinical diagnosis* of FXS without relying on any genetic or familial data.

**Conclusion:**

Although FXS is often thought of primarily as a neurological disorder, it is in fact a multisystem syndrome involving many co-occurring conditions, some primary and some secondary, and they are associated with a considerable burden on patients and their families.

## INTRODUCTION

Fragile X syndrome (FXS) is the most common inherited cause of intellectual disability and autism.^1^ It has no evident phenotype detectable at birth and the physical characteristics are subtle and nonspecific.^2,3^ Genetic testing is recommended when speech and developmental delays become evident.^3,4^ Diagnosis often is the result of cascade testing after a child in the family is diagnosed with FXS.^5,6^ Early diagnosis of this inherited syndrome has important implications for patients and families and would allow for timely intervention, appropriate genetic counseling, and family planning.^3,7,8^ However, despite increased emphasis on identification of individuals with disabilities, patient advocacy efforts, and accessibility of genetic testing, FXS remains significantly underdiagnosed.^9^

Past research has shown that the diagnostic odyssey ending in genetic testing for FXS in children takes 18 to 24 months after parents express concerns, resulting in otherwise avoidable stress on families and gaps in service provision to the child who ultimately receives a diagnosis of FXS.^2,6^ In many cases families have a second child with FXS before receiving the diagnosis for the first child, which underscores the importance of early diagnosis in children, particularly in the first decade of life.^6^ Additionally, identification of an individual with FXS can lead to cascade testing throughout the family. It can result in identification of other family members who also have the full mutation or a premutation. Recent studies have shown that individuals with a premutation could experience a wide range of medical problems including fragile X–associated primary ovarian insufficiency (FXPOI), fragile X–associated tremor/ataxia syndrome (FXTAS), and other disorders.^10–12^ Additionally, females with a premutation are at risk of having children with FXS.^13^ Recent advancement in clinical trials of targeted treatment and the benefit of early intervention have increased the interest in newborn screening for this condition.^14,15^ However, currently it does not meet the criteria for newborn screening. Furthermore there is a need to develop other methods to identify those who potentially might have the condition and have not been diagnosed.

Comprehensive evaluation of genotype–phenotype associations in FXS offers a potential pathway to earlier diagnosis and effective intervention. Clinical studies have shown that individuals with FXS have a complex health profile leading to unique clinical needs.^3,16–18^ Therefore knowledge of the full phenotypic manifestation of the disease will improve multiple aspects of patients’ health. In addition to accelerating the diagnosis, such knowledge will inform public health policies regarding services needed by families and patients. Some behavioral problems in these individuals could be the result of pain from an undiagnosed medical condition.^3^ Thus, diagnosis and treatment of secondary conditions could also improve primary outcomes in these patients.

Identification of the full spectrum of lifetime medical conditions associated with FXS remains challenging. The phenotypic manifestation of this X-linked disorder is variable depending on age, sex, and molecular variations.^3^ It often includes symptoms of social anxiety,^19^ intellectual and learning disability,^20^ behavioral problems,^21^ attention-deficit/hyperactivity disorder (ADHD),^22^ sleep difficulties,^23^ language deficits,^24^ motor problems,^17^ sensory integration challenges,^25^ and seizures.^17,26^

Additionally several clinical studies have reported medical issues such as recurrent otitis media and recurrent sinusitis, joint laxity and pes planus (flat feet), gastroesophageal reflux disease, and mitral valve prolapse^3^ in children. However, the prevalence of these medical issues in these individuals has not yet been estimated using population data, nor has the difference in prevalence between those with FXS and those in the general population been investigated.

In recent years, the Fragile X Clinic and Research Consortium (FXCRC) has facilitated data collection from patients receiving care from specialized FXS clinics, providing critical information about the health of such individuals. However, data collected in these clinics could potentially overrepresent individuals whose families have the resources or opportunity to access premier care. Behavioral challenges in the most seriously affected patients might prevent the families from traveling to specialty clinics; thus they may not be included in the Fragile X Online Registry With Accessible Research Database (FORWARD). Importantly, although the FXCRC collects data from family members with fragile X–associated disorders, an independent control group representing the general population is not available through FORWARD. The majority of FXS cases in FORWARD (>90%) are younger than age 25; thus the full extent of the impact of FXS on adults and aging patients is not characterized in this database.^27^ Investigation of the complete medical history of FXS patients obtained from a population-based data set can complement the information contained in FORWARD.

The digitization of patient medical data has created an unprecedented opportunity to rapidly ascertain comprehensive, multidimensional, and population-level clinical data.^28^ The availability of big biomedical data in conjunction with advancements in artificial intelligence (AI) has the potential to transform current clinical practice, and improve disease risk evaluation and guide intervention plans.

Here, we report on our efforts to mine the electronic health records (EHRs) from more than one million people (all of whom were served by a single health-care system), to investigate the health characteristics of individuals clinically diagnosed with FXS. Our investigation proceeded in three phases. First, we focused on examining the impact of the disease on *lifetime physical health conditions* reported in individuals with clinically diagnosed FXS. Second, we examined *mental and neurological conditions* to report differences between cases and controls in the prevalence of these co-occurring conditions using unbiased population-level data. Third, we created predictive models based on EHR entries recorded at least *five years prior to clinical diagnosis of FXS without relying on any genetic data*. We developed a timeline of key conditions that had been entered into the EHRs at least five years prior to the clinical diagnosis of FXS and showed that our AI-assisted diagnosis can be instrumental in accelerating the identification of undiagnosed cases in the general population.

## MATERIALS AND METHODS

### Study population

We examined the de-identified EHRs from 1,723,223 patients (802,832 males, 920,385 females, 6 unknown) who received care from the Marshfield Clinic health-care system, a large not-for-profit, multispecialty health-care system, serving patients from Northern and Central Wisconsin. Marshfield Clinic was one of the first US institutions to develop an EHR system, which now includes approximately 40 years (1979–2018) of continuous and virtually comprehensive health data for average participants. According to this data, 1,301,358 patients (620,109 males, 681,248 females, and 1 unknown) had three or more medical encounters. The remaining patients had fewer than three encounters with the clinic and their records were excluded from the current study. The diagnoses in the EHR recorded prior to 2015 are coded in the form of the International Classification of Diseases,^29^ Ninth Revision (ICD-9) codes and since then the International Classification of Diseases, Tenth Revision (ICD-10) have been adopted in the Marshfield Clinic. We mapped all of the diagnoses to the ICD-9 codes to harmonize the data set. To further prepare the data, we restricted the analyses to codes that appeared at least twice for a given participant (rule of 2), and that were observed in at least five individuals. The rule of 2 is a well-established approach in analyzing EHR data. It indicates that at least 2 independent pieces of evidence are required for inclusion of a condition in the analysis. Rule of 2 reduces the chance that tests used to rule out a condition (i.e., testing recommendation without a positive outcome) are misinterpreted to be the presence of the condition and it can improve the accuracy of the classification.^30^

### Case and control identification

We identified 82 participants who received a diagnostic code for FXS (ICD-9 = 759.83) in their medical records. We limited our cases to the ones who received the code on at least two occasions (rule of 2) resulting in 55 cases (11 females, 44 males). The diagnostic codes of the 27 individuals who received a FXS diagnosis only one time were reviewed by a physician (D.S.). The summary of these medical records is reported in the Supplementary materials justifying their exclusion from the current study.

To represent the general population, the only exclusion criterion for controls was receiving a diagnosis of FXS. From potential controls, we selected 5,500 participants (1 to 100 ratio) who matched cases on sex and year of birth (Table S1).

### Random forest classifier

Random forest is a nonlinear classifier capable of processing high dimensional data with low generalization error and high predictive performance.^31^ It can discover important multivariate interactions in the data and enable us to find predictive combinations of diagnostic codes that differentiate the two groups. This method can be successfully applied to skewed data sets with unbalanced number of cases and controls that have large numbers of input variables compared with the sample size.^31^ To evaluate the classifier success area under receiver operating characteristic curve (AUROC) is reported. Tenfold cross-validation was applied to ensure that the ROC curve is not overly optimistic. To measure whether the classifier is performing significantly better than random (AUROC = 0.5), we used the Mann–Whitney–Wilcoxon test (Mann–Whitney *U*-test). To identify variables contributing in the predictive model, we used a measure called mean decrease in impurity based on Gini (MDG) coefficient. The detailed description of these approaches is provided in the Supplementary materials.

### Mapping diagnostic codes to clinical phenotypes

We used phenome-wide association study (PheWAS) approaches to examine the phenotypic association of clinical diagnoses and FXS.^12,32^ We mapped the ICD-9 codes to clinical phenotypes (phecodes) using the PheWAS mapping function developed in R.^32^ A total of 7,122 unique diagnostic codes were extracted from the EHRs of these participants (those with a FXS diagnosis and controls), which later mapped in to 1,203 phecodes. To examine the health characteristics of participants, we used the total frequency of phecodes as our input variable and performed linear regression. We also examined the presence and absence of the phecode as an alternative approach and used logistic regression to identify possible associations and reported the odds ratio.

## RESULTS

### Phase I: lifetime physical health conditions

**Fig. 1:**
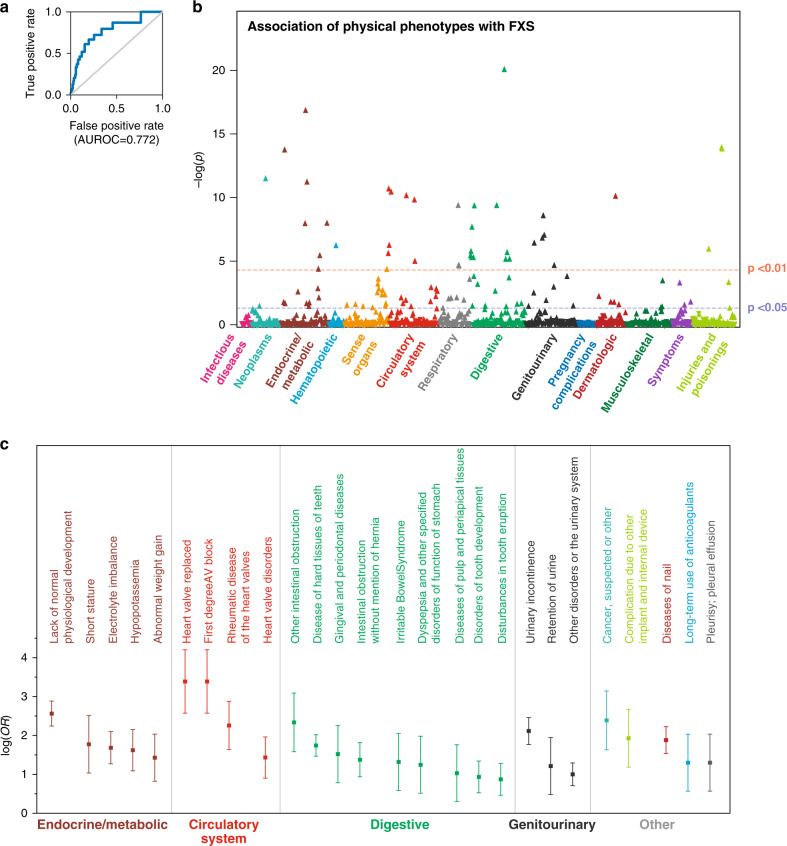
Physical health conditions of individuals with fragile X syndrome (FXS). **a** Receiver operating characteristic curve of classifier performance identifying individuals with FXS using physical health conditions. Area under the receiver operating characteristic curve (AUROC) shows there are significant differences between physical health conditions of patients with FXS and the general population (*p* value = 1.3e-13). **b** Manhattan plots of unadjusted −log_10_ (*P* values) for phenotypes observed in individuals with FXS. Each point shows one phenotype. Blue line shows the significance threshold (*p* < 0.05), all phenotype above the line are associated with FXS. Red line shows the threshold for multiple comparisons adjustment (*p* < 0.00005). All phenotypes above the red line survived adjustments for multiple comparisons (39 codes). **c** Odds ratio and 95% confidence interval for phenotypes associated with FXS in multiple cases (26 codes) that survived adjustment for multiple comparisons.

The goal of this first phase of the analysis was to examine the impact of FXS on patients beyond known co-occurring conditions. We removed phenotypes that are directly related to FXS (i.e., mental health, neurological disorders, congenital anomalies) and instead focused on less-explored phenotypic categories (i.e., physical health conditions). All 55 cases and age–sex matched controls were included in this phase of the analysis. By implementing a random forest classifier developed on only physical health conditions (Fig. 1a, Fig. S1), we were able to successfully differentiate individuals with FXS from controls (AUROC = 0.772, *p* value = 1.3e-13) meaning that there is a significant difference between the physical health conditions of cases with FXS compared with the general population and that the impact of FXS is beyond mental and neurological conditions.

Next, we used a PheWAS approach to further examine the phenotypic association of physical health conditions and FXS. One hundred significant associations (*p* < 0.05) were identified from which 39 survived adjustments for multiple comparisons using Bonferroni corrections (Fig. 1b). These phenotypes include disorders associated with the circulatory system, digestive system, endocrine and metabolic disorders, respiratory problems, genitourinary conditions, and others.

After identifying physical health conditions significantly associated with FXS, we examined the participants’ EHRs in terms of two indicators of the burden of disease:^12^ (1) the percentage of cases and controls who received a diagnosis for each phenotype, and (2) the number of medical encounters for each condition for cases versus controls (Table 1). The odds ratios and 95% confidence intervals for these conditions are shown in Fig. 1c. We observed that individuals with FXS had a higher number of medical encounters for circulatory disorders. For example, heart valve disorders were five times more frequently recorded in the EHRs of FXS cases compared with the general population. The average number of medical encounters for this diagnosis was 25.4 versus 12.5 for the controls and the median age of receiving this diagnosis in cases was 26 compared with 52 in controls. Individuals with FXS had multiple conditions related to the digestive system including intestinal obstruction, dyspepsia, other disorders of the function of stomach, and irritable bowel syndrome. FXS cases generally were diagnosed with digestive problems at a younger age compared with the controls. For example the median age of diagnosis with intestinal obstruction in FXS cases was 37 compared with 60 in controls.

**Table 1:** Physical health conditions associated with fragile X syndrome (FXS).

	FXS (*n* = 55)		Controls (*n* = 5,500)		
Description	Prevalence (percentage)	Average encounters	Prevalence (percentage)	Average encounters	*P* value (encounters)
**Circulatory**					
Heart valve replaced	3.64	14	0.13	11	4.01E-11
First degree atrioventricular (AV) block	5.45	3.33	0.13	4.14	7.71E-11
Heart valve disorders	9.09	25.4	1.85	12.5	6.29E-07
Rheumatic disease of the heart valves	7.27	5.5	0.6	5.45	2.76E-06
**Digestive**					
Dyspepsia and other specified disorders of function of stomach	5.45	7.33	1.09	3.07	4.66E-10
Other intestinal obstruction	3.64	24.5	0.38	13	2.37E-06
Intestinal obstruction without mention of hernia	3.64	27.5	0.82	9.96	7.98E-06
Irritable bowel syndrome	3.64	7.5	1	3.84	9.57E-06
**Dental**					
Gingival and periodontal diseases	10.91	4.83	3	2.65	5.34E-10
Diseases of hard tissues of teeth	43.64	5	11.93	4.72	2.47E-08
Disorders of tooth development	12.73	3.57	5.75	2.42	4.51E-06
Diseases of pulp and periapical tissues	5.45	4	1.33	2.67	5.92E-06
Disturbances in tooth eruption	12.73	3.14	5.42	2.31	6.59E-06
**Endocrine/metabolic**					
Short stature	3.64	21.5	0.64	5.89	1.51E-17
Abnormal weight gain	7.27	5	1.38	3.04	1.16E-08
Lack of normal physiological development	27.27	7.93	2.58	7.74	1.24E-08
Hypokalemia	9.09	7.2	1.53	5.6	4.05E-06
Electrolyte imbalance	12.73	9	2.64	6.99	4.74E-05
**Genitourinary**					
Retention of urine	3.64	22	1.11	5.7	2.94E-09
Urinary incontinence	20	7.09	2.93	6.23	1.01E-07
Other symptoms/disorders or the urinary system	30.91	9.71	14.13	5.47	1.80E-07
**Other conditions**					
Diseases of nail	20	5.45	3.69	4.22	8.51E-11
Encounter for long-term (current) use of anticoagulants	5.45	76	1.02	32.46	6.71E-07
Complication due to other implant and internal device	3.64	5.5	0.55	3.1	1.26E-06
Cancer, suspected or other	3.64	30	0.35	8.79	3.60E-12
Pleurisy; pleural effusion	3.64	21	1.02	7.45	2.74E-05

Conditions that were observed in two or more cases and survived adjustments for multiple comparisons are listed.

Dental problems also were more common in FXS cases with 43.64% compared with 11.93% in controls. Additionally FXS cases had higher rates of endocrine and metabolic disorders. For example 12.73% had electrolyte imbalance compared with 2.64% in controls. Genitourinary problems such as urinary incontinence were also frequently reported in FXS cases. Significant differences were observed for 61 other conditions that did not survive adjustment for multiple comparisons. The complete list of physical health conditions associated with FXS is shown in Table S2. To provide sex-specific results, in a follow-up analysis we examined the physical health characteristics of males with FXS (Table S3). Similar associations to the full patient cohort were observed in males with FXS.

### Phase II: co-occurring mental and neurological conditions and congenital anomalies

In this second phase of the analysis, we shifted focus to the conditions most frequently described in individuals with FXS, and investigated the differences between diagnosed cases and controls in the prevalence of these conditions. The results yielded 41 conditions that significantly differentiated the two groups (*p* < 0.05), of which 28 survived Bonferroni adjustments for multiple comparisons (Table 2). Similar to phase I, all 55 cases and age–sex matched controls were included in this phase of the analysis.

**Table 2:** Mental and neurological disorders associated with fragile X syndrome (FXS).

	FXS (*n* = 55)		Controls (*n* = 5,500)		
Description	Prevalence (percentage)	Average encounters	Prevalence (percentage)	Average encounters	*P* value (encounters)
**Mental disorders**					
Specific nonpsychotic mental disorders due to brain damage	34.55	12.58	1.85	8.59	2.82E-73
Other specified nonpsychotic and/or transient mental disorders	38.18	12.71	3.45	7.06	4.01E-68
Autism	25.45	113.71	1.07	39.32	6.96E-37
Pervasive developmental disorders	58.18	69.72	12.87	21.51	2.41E-34
Generalized anxiety disorder	5.45	51.67	2.09	9.52	4.78E-32
Psychosis	7.27	14.75	0.95	6.54	3.32E-29
Speech and language disorder	23.64	6.15	2.24	5.98	6.32E-29
Altered mental status	5.45	8.33	0.67	3.86	1.08E-24
Impulse control disorder	10.91	16.5	0.91	13.02	1.60E-16
Attention-deficit hyperactivity disorder	49.09	21.85	11.53	18.8	4.84E-16
Developmental delays and disorders	67.27	23.08	4.75	15.02	8.34E-12
Conduct disorders	45.45	15.68	5.84	15.12	1.10E-10
Anxiety, phobic, and dissociative disorders	49.09	26.41	15.35	15.6	1.40E-10
Intellectual disability	49.09	25.93	0.76	48.21	4.97E-10
Neurological disorders	34.55	9.79	4.42	8.74	1.70E-09
Schizophrenia and other psychotic disorders	12.73	33.29	1.33	28.96	2.69E-09
Aphasia/speech disturbance	25.45	10.79	2.15	12.81	2.27E-08
Anxiety disorder	12.73	26.43	4.98	10.62	2.51E-08
Other persistent mental disorders due to conditions classified elsewhere	14.55	3.88	0.8	6.68	2.95E-08
Alteration of consciousness	9.09	5	1.69	4.54	4.04E-08
Schizophrenia	5.45	39.67	0.6	41.91	5.99E-06
**Neurological disorders**					
Epilepsy, recurrent seizures, convulsions	23.64	30.85	3.07	17.91	3.15E-15
Convulsions	18.18	12.4	2.35	8.25	3.49E-13
Partial epilepsy	3.64	45	0.75	17.8	6.82E-07
Epilepsy	3.64	55	1.24	19.54	1.99E-05
**Congenital anomalies**					
Chromosomal anomalies and genetic disorders	100	20.4	0.51	28.96	3.48E-188

All of the conditions survived adjustments for multiple comparisons.

Focusing on mental and neurological disorders (Table 2), we observed that 25.45% of individuals with FXS also received a diagnosis for autism compared with 1.07% in the general population. The average number of medical encounters in cases for this diagnosis was 113.71 versus 39.32 visits in controls. Developmental delays and disorders (67.27% vs. 4.75%); ADHD (49.09% vs. 11.53%); aphasia/speech disturbance (25.45% vs. 2.15%); schizophrenia and other psychotic disorders (12.73% vs. 1.33%); anxiety, phobic, and dissociative disorders (49.09% vs. 15.35%); and epilepsy, recurrent seizures, and convulsions (23.64% vs. 3.07%) were more common in individuals with FXS. In almost all of these diagnoses, the average number of medical encounters was higher in the FXS group.

Chromosomal anomalies and genetic disorders were reported in both cases and controls (Table 2). All cases (100%) received this phecode due to having the FXS diagnosis. Furthermore, 28 individuals (0.5%) in the control group had disorders such as Down syndrome, Edwards syndrome, Marfan syndrome, Klinefelter syndrome, gonadal dysgenesis, Prader–Willi syndrome, and velocardiofacial syndrome. Table S4 shows the complete list of mental and neurological phenotypes that were significantly associated with FXS and Table S5 lists the result of the subsequent analysis focused on males.

### Phase III: predictive model to identify FXS status five years prior to clinical diagnosis

**Fig. 2:**
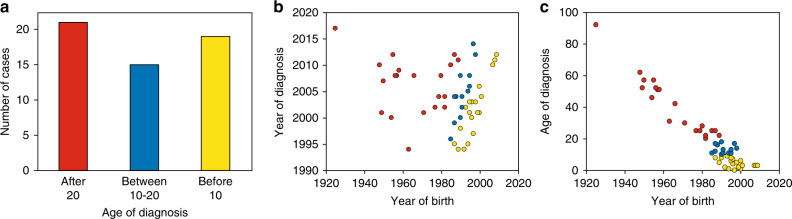
Age of diagnosis in fragile X syndrome (FXS) cases. **a** Distribution of age of diagnosis. **b** Association between year of birth and year of diagnosis. In this population, there is no association between year of birth and year of diagnosis. **c** Association between year of birth and age of diagnosis. The age when the diagnosis of FXS was first reported in the electronic health records (EHRs) varies widely in this community patient population, ranging from 6 months to 92 years of age. The age of diagnosis has decreased in more recently born cases.

The goal of this third phase of our study was to determine the possibility of prescreening to identify individuals as potentially having FXS five years in advance of their diagnosis of FXS by using entries in their EHRs. The age when the diagnosis of FXS was first reported in the EHRs varies widely in this community patient population, ranging from 6 months to 92 years of age (Fig. 2). Genetic testing for FXS became available in 1991^33^ and the first case in the Marshfield Clinic was diagnosed in 1994. In this population, there is no association between year of birth and year of diagnosis (Fig. 2b). However, the age of diagnosis has decreased in more recently born cases (Fig. 2c).

To identify underdiagnosed cases who were not identified in early childhood, we created a predictive model focusing on the individuals who received the diagnosis at age 10 or older. Thirty-six cases met this criterion (6 females and 30 males). We applied a case–control matching approach (discussed in “Materials and Methods”) and selected 3,600 controls for the analysis. A random forest classifier was created using only the diagnostic codes entered into the EHRs *at least five years prior to receiving a diagnosis for FXS* in cases. For matched controls, the codes that were entered into the EHRs during a similar duration of care were included in the analysis. The model was created blind to any prior knowledge about FXS phenotypes and no priority was given to any co-occurring conditions discussed in phases I and II of current study or phenotypes previously reported in the literature.

**Fig. 3:**
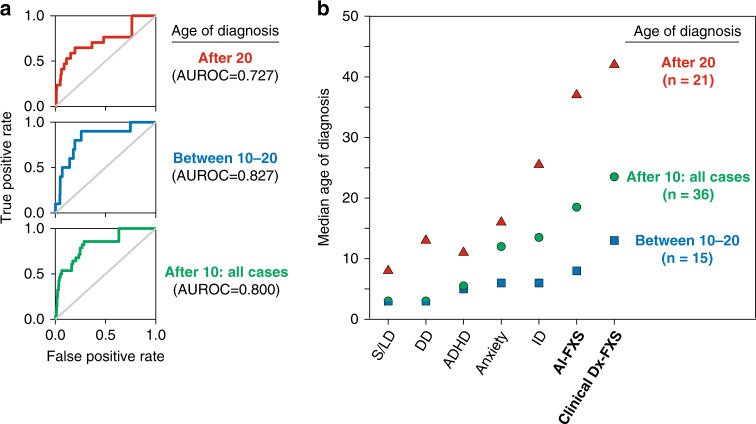
Artificial intelligence–assisted diagnosis. **a** Receiver operating characteristic curve of classifier performances identifying individuals with fragile X syndrome (FXS) using their electronic health record (EHR) data five years prior to receiving clinical diagnosis. Cases and controls are matched on sex and year of birth with 1:100 ratio. **b** Timeline of median age of diagnosis for key conditions associated with FXS. ADHD attention-deficit hyperactivity disorder, AI-FXS artificial intelligence–assisted prediction of FXS diagnosis, Clinical Dx-FXS clinical diagnosis of FXS as reported in the medical report, DD developmental delay, ID intellectual disability, S/LD speech and language disorders. Our artificial intelligence (AI)-assisted approach is able to identify cases five years earlier than the time of clinical diagnosis.

We created three separate models: (1) the subset of cases who received the diagnosis between ages 10 and 20, (2) the subset of cases who were diagnosed after age 20, (3) all cases diagnosed after age 10. In all three models, we were able to successfully identify cases from controls five years prior to the time when each received a clinical diagnosis, with AUROCs of 0.827, *p* = 6.9e-05; 0.727, *p* = 0.0003; and 0.800, *p* = 6.2e-11, respectively (see Fig. 3a, Fig. S2).

Examination of the cumulative model, using the mean decrease in Gini coefficient (Table S6), showed that individuals with FXS received diagnostic codes for intellectual disability, anxiety disorder, ADHD, depressive disorder, acute upper respiratory infections, acute pharyngitis, unspecified disorder of the teeth and supporting structures, lack of normal physiological development, and unspecified otitis media, *at least five years prior to being diagnosed with FXS*.

We next created a timeline depicting the order and median age of being diagnosed with prior conditions, based on these 36 cases (Fig. 3b). The overall timeline shows that these individuals were diagnosed with developmental delay and speech/language disorder at a median age of 3, ADHD at age 5.5, anxiety disorder at 12, and intellectual disability at 13.5. However, they did not receive the FXS diagnosis until the median age of 23.5 years old. Thus, our predictive model is able to accelerate identification of these underdiagnosed cases in the population.

## DISCUSSION

Our discovery-oriented approach investigates the health characteristics of individuals with FXS based on population data incorporating the entire spectrum of available health diagnoses. Our analysis confirms the ability of computational methods to identify phenotypes associated with a target genetic condition using medical codes in the EHRs. Our results provide evidence of FXS association with a wide range of physical health conditions.

We found an alarming rate of serious heart disorders in FXS patients. General heart problems including mitral valve prolapse (MVP), heart rhythm disorder, cardiac conduction abnormalities, and heart attack have been previously reported in the clinical literature^17^ but their prevalence has not been reported. It is speculated that loose connective tissue and abnormalities of elastin fibers can cause MVP and weakening of vessels in the form of aortic dilatation in these individuals.^3^ Our results confirm that regular screening for circulatory disease is critical for FXS patients.

We also observed conditions such as short stature and abnormal weight gain. Abnormal weight has been frequently reported in adults with FXS.^17^ Impaired hypothalamic functioning in individuals with FXS due to decreased levels or absence of fragile X mental retardation protein (FMRP) could be a potential cause of abnormal weight gain in these individuals.^3^ Additionally, increased appetite is a common side effect of the psychotropic medications that are frequently used in patients with FXS.^34^

Long-term use of medications can also cause electrolyte imbalance and hypokalemia in the patients. Electrolyte imbalance could cause other medical problems such as fatigue, muscle weakness, and high blood pressure. Many of these conditions are reversible if effectively managed at an early stage.^35^ Further examination of medication history is required to clarify the nature of endocrine and metabolic disorders in these patients. Structural issues related to facial morphology (long face and a high-arched palate) and loose connective tissue could be a potential reason for higher rate of dental and gum problems in FXS cases.^36^ Additionally, behavioral problems in these individuals might lead to barriers to receiving proper dental care. Some of the dental disorders could also be related to medication side effects.

We found consistent evidence of higher rates of mental and neurological conditions such as developmental delay, anxiety, speech problems, and epilepsy in individuals with FXS. More than half were diagnosed with pervasive developmental disorders and 25.45% were diagnosed with autism. This is consistent with previously reported rates^37^ of these conditions in patients with FXS and reinforces the recommendation by the American Academy of Pediatrics,^38^ the American Academy of Neurology, and Child Neurology Society^39^ to consider FXS testing in patients with autism, global developmental delay, or intellectual disability.

By using artificial intelligence approaches, we successfully created predictive models to identify cases at least five years earlier than the time of clinical diagnosis of FXS without using any genetic or family history information. The timeline of key co-occurring conditions in medical records can provide critical information for physicians about the manifestation of phenotypes in FXS cases and reduce the time of clinical diagnosis.

What is the significance of these findings? Our discovery-oriented approach in mining population-based medical data results in an unbiased evaluation of health in individuals with FXS. Our results indicate that although FXS is often thought of primarily as a neurological disorder, it is in fact a multisystem syndrome involving many co-occurring conditions, some primary and some secondary, and they result in a considerable burden on patients and their families. Incorporation of artificial intelligence approaches into the medical system could serve as a prescreening tool and create a structure to automatically alert physicians about the presence of multiple FXS-related phenotypes in the patient’s medical records. By prompting the physician to further evaluate such individuals and refer them for genetic testing and counseling, our approach could accelerate the diagnostic process and be instrumental in identifying undiagnosed adults in the population and addressing their health conditions.

There are limitations to the current study that should be noted. Although a large population of patients was mined, the number of clinically diagnosed cases was small, particularly for the females. Additionally, the predictive model is trained only on individuals diagnosed in the second decade of life (age 10 or older). A larger number of cases is needed to investigate the genotype–phenotype associations reported here and to develop age-specific models to identify potential cases in early childhood. Furthermore, an independent data set is required to evaluate the performance of our approach in identifying potential cases.

Females with FXS experience increased variability in symptoms and are often more mildly affected when compared with males due to the presence of the second X chromosome. The milder level of symptoms could contribute to the higher rate of underdiagnosis of FXS among females. The current study includes almost four times more males than females and the phenotypes reported in this cohort might be more driven by male cases than females.

The Marshfield population is relatively homogeneous with the vast majority of participants reporting themselves to be Northern European/White Caucasian.^40^ Therefore, the lack of diversity is a limitation of the current study and additional studies are required to examine the presence of identified phenotypes in other populations.

Providing timely diagnosis and intervention for FXS is an important public health goal. Understanding the complicated health profile of FXS and its implications for the health and well-being of patients and their families will improve current clinical practice and the quality of life of these individuals. Our AI-assisted approach can support health-care providers in identifying individuals with FXS and facilitate timely response to the unique clinical needs of patients with FXS in a multidisciplinary setting.

## Supplementary Information

Supplementary Materials

## Data Availability

All data needed to evaluate the conclusions in the paper are presented in the paper or in the Supplementary materials. De-identified data can be requested from the Marshfield Clinic. For more information contact the Research Compliance Office at +1-715-221-7040.
