# Supplementary material for: Artificial intelligence–assisted phenotype discovery of fragile X syndrome in a population-based sample
Source: Genet Med. 2021 Mar 26;23(7):1273–80. doi: 10.1038/s41436-021-01144-7 (PMC8257481; doi:10.1038/s41436-021-01144-7)
Supplement: Supplementary file 1 — Supplementary Materials [file 41436_2021_1144_MOESM1_ESM.doc]

Supplementary Materials for

**Artificial Intelligence-Assisted Phenotype Discovery of Fragile X Syndrome in a Population-Based Sample**

Arezoo Movaghar, David Page, Danielle Scholze, Jinkuk Hong, Leann Smith DaWalt, Finn Kuusisto, Ron Stewart, Murray Brilliant, Marsha Mailick^*^

*Correspondence to: [marsha.mailick@wisc.edu](mailto:marsha.mailick@wisc.edu)

**Exclusion Based on Rule of two**

We reviewed the diagnostic codes of the 27 individuals (18 males, 9 females, median age=26) who received a fragile X syndrome (FXS) diagnosis only one time and have been excluded from the study. The following was observed: 1) Some of these individuals (n=8) were diagnosed with other congenital anomalies (e.g., Congenital hydrocephalus, Klippel-Feil syndrome, and Marfan syndrome) and were possibly tested for fragile X syndrome as part of the diagnostic process. 2) Several female patients (n=5) had conditions such as menstrual and reproductive issues. The testing was recommended to investigate their premutation status. 3) Others (n=7) had conditions such as developmental delay, ADHD and speech delay which could be the reason for genetic referral in these individuals. However there is no indication of a positive genetic result in the records. 4) 7 cases had a limited number of encounters (less than 3 visits) and therefore sufficient data is not available for evaluation. By applying the rule of 2 to identify the potential positive FXS cases, we were able to eliminate individuals who were tested for FXS without further evidence of a positive clinical diagnosis (likely ruling out FXS).

**Prevalence estimate of FXS in the Marshfield population**

Marshfield Clinic data includes de-identified electronic health records from 1,723,223 patients (802,832 males, 920,385 females, 6 unknown). 1,301,358 patients (620,109 males, 681,248 females and 1 unknown) had 3 or more medical encounters with the clinic. The remaining patients had less than 3 encounters and therefore their records were excluded from the current study.

The mean prevalence of FXS based on population based genetic testing is estimated to be 1.4 per 10,000 in males (or 1/7,143) and 0.9 per 10,000 in females (or 1/11,111)^1^. The number of individuals with FXS in Marshfield population is estimated to be 149 (87 males:$\left\lceil620,109 /7,143 \right\rceil$ and 62 females: $\left\lceil681,248/11,111 \right\rceil$). Therefore there is a difference between the number of individuals who actually receive the diagnosis through the medical system and prevalence estimates based on population genetic testing (p-value=9.278e-15). Genetic testing of the entire population would be required to identify the exact number of FXS cases, which is well beyond the scope of the present research. One of the main goals of the present study is to develop predictive models to facilitate identification of under-diagnosed cases in the population.

**Random forest classifier**

Random forest is an ensemble of decision trees. The algorithm operates by repeatedly drawing a bootstrap sample (random sampling with replacement) from the data and constructing a tree for each bootstrap sample^2^. A decision tree classifies samples by posing a series of tests on the variables associated with the samples^3^. Random forest consists of a collection of such trees. In this study we used classifiers with 1000 trees. At each node of the tree, only a random subset of variables is considered for training, which increases the accuracy of classification, decreases the sensitivity to noise in the data, and minimizes the correlation among the variables^2^. The number of random variables (k) is equal to int(log_2_(number of input variables) +1)). The ensemble nature of the method reduces the possibility of over-fitting. Each tree makes an independent prediction. The majority of the predictions aggregated from all trees determines whether a case should be classified as FXS or control^2^.

To measure the success of classification, the area under receiver operating characteristic curve (AUROC) is reported^4^. The ROC curve shows the false-positive rate versus the true-positive rate resulting from the classification task. AUROC of 1.00 shows 100% success in classification meaning that classifier was able to successfully assign all of the cases to the correct class and AUROC of 0.5 represents random classification. Ten-fold cross validation was used to ensure that the ROC curve is not overly optimistic. Participants were randomly partitioned into ten parts and the learning algorithm was applied 10 times. In each run, a different part of participants were held aside as the test set and the predictive model was trained on the remaining nine-tenths of the data and then tested on the held-out one-tenth. The ROC curve is constructed from the test set and the ten final curves are averaged and reported. To measure whether the classifier is performing significantly better than random (AUROC = 0.5), we used the Mann-Whitney-Wilcoxon test (Mann-Whitney U test). Because a single ROC curve is generated, adjustment for multiple comparisons is not necessary^4,5^.

To identify variables contributing in the predictive model, we used a measure called *mean decrease in impurity based on Gini* (MDG) coefficient. MDG indicates how often a particular variable (i.e. diagnostic code) was selected for a split, and what was the overall discriminative value of that variable for classification. The best variables tend to split mixed class nodes (FXS and controls) into pure single class (FXS or controls) nodes. Variables with higher MDGs are more influential in creating decision trees for prediction^6–8^.


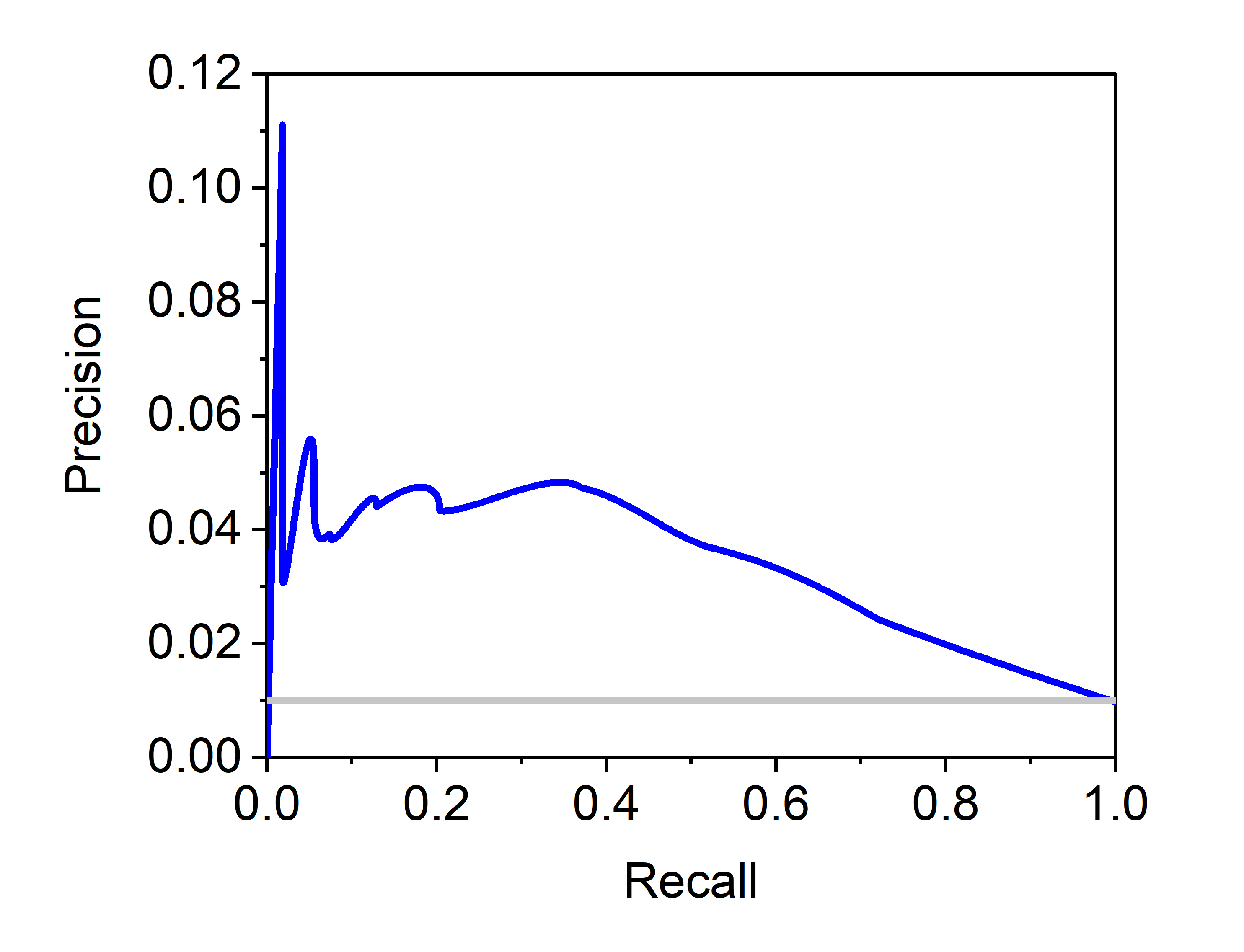


**Figure S1. Precision-Recall curve of classifier performance identifying individuals with FXS using physical health conditions.** Cases and controls are matched on sex and year of birth with 1:100 ratio. Therefore the AUPRC for the baseline classifier is equal to 0.0099. Area under precision recall curve (AUPRC) for the random forest classifier was 0.034.

**Figure S2. Precision-Recall curve of classifier performances identifying individuals with FXS using their EHR data five years prior to receiving clinical diagnosis.** Area under precision recall curve (AUPRC) is reported for each classifier. Cases and controls are matched on sex and year of birth with 1:100 ratio, therefore the AUPRC for the baseline classifier is equal to 0.0099. **A)** Cases diagnosed after age 20 (number of cases=20) with AUPRC=0.054. **B)** Cases diagnosed between age 10-20 (number of cases=16) with AUPRC=0.052. **C)** Cases diagnosed after age 10 (number of cases=36) with AUPRC=0.183.

**Table S1. Participants’ characteristics.** FXS cases and controls were matched on year of birth and sex.

|  | **FXS cases (n=55)** | | **Controls (n=5500)** | |
| --- | --- | --- | --- | --- |
|  | Range | Median | Range | Median |
| Year of birth | 1925-2009 | 1989.5 | 1925-2009 | 1989.5 |
| Age of FXS diagnosis (years) | 0.5-72 | 13 | - | - |
| Total number of codes | 16-2384 | 268 | 5-5691 | 74 |
| Number of unique codes | 8-249 | 67 | 1-481 | 31 |

**Table S2. Physical health conditions associated with FXS.** Conditions with significant p-value (<0.05) that were observed in 2 or more cases are listed. Health categories are color coded as follows: circulatory (pink), digestive (green), dental problems (light gray) endocrine/metabolic (orange), genitourinary (white), disease of sense organs (purple), other conditions (gray).

|  | **FXS (n=55)** | | **Controls (n=5500)** | |  |
| --- | --- | --- | --- | --- | --- |
| Description | Prevalence  (Percentage) | Average encounters | Prevalence  (Percentage) | Average encounters | P-value  (Encounters) |
| Heart valve replaced | 3.64 | 14 | 0.13 | 11 | 4.01E-11 |
| First degree AV block | 5.45 | 3.33 | 0.13 | 4.14 | 7.71E-11 |
| Heart valve disorders | 9.09 | 25.4 | 1.85 | 12.5 | 6.29E-07 |
| Rheumatic disease of the heart valves | 7.27 | 5.5 | 0.6 | 5.45 | 2.76E-06 |
| Hypotension | 5.45 | 8 | 1.42 | 5.22 | 2.63E-03 |
| Encounter for long-term (current) use of anticoagulants, antithrombotics, aspirin | 5.45 | 4.33 | 0.38 | 6.29 | 6.73E-03 |
| Nonspecific chest pain | 10.91 | 21.17 | 12.49 | 6.79 | 8.70E-03 |
| Atrioventricular [AV] block | 5.45 | 3.33 | 0.33 | 5.44 | 1.36E-02 |
| Cardiac dysrhythmias | 7.27 | 42.25 | 6.87 | 14.24 | 4.26E-02 |
| Dyspepsia and other specified disorders of function of stomach | 5.45 | 7.33 | 1.09 | 3.07 | 4.66E-10 |
| Other intestinal obstruction | 3.64 | 24.5 | 0.38 | 13 | 2.37E-06 |
| Intestinal obstruction without mention of hernia | 3.64 | 27.5 | 0.82 | 9.96 | 7.98E-06 |
| Irritable Bowel Syndrome | 3.64 | 7.5 | 0.96 | 3.87 | 9.57E-06 |
| Functional digestive disorders | 20 | 7.18 | 6.16 | 5.1 | 2.38E-04 |
| Disorders of function of stomach | 7.27 | 6.5 | 1.29 | 4.73 | 2.58E-03 |
| Abnormal serum enzyme levels | 5.45 | 7.33 | 1.64 | 5.6 | 2.83E-02 |
| Diseases of lips | 3.64 | 2 | 0.45 | 2.88 | 3.86E-02 |
| Paralytic ileus | 3.64 | 3 | 0.35 | 5.26 | 4.36E-02 |
| Gingival and periodontal diseases | 10.91 | 4.83 | 2.98 | 2.65 | 5.34E-10 |
| Diseases of hard tissues of teeth | 43.64 | 5 | 11.85 | 4.73 | 2.47E-08 |
| Disorders of tooth development | 12.73 | 3.57 | 5.69 | 2.42 | 4.51E-06 |
| Diseases of pulp and periapical tissues | 5.45 | 4 | 1.33 | 2.67 | 5.92E-06 |
| Disturbances in tooth eruption | 12.73 | 3.14 | 5.36 | 2.31 | 6.59E-06 |
| Periapical abscess | 3.64 | 4.5 | 1.07 | 2.69 | 1.82E-04 |
| Other diseases of the teeth and supporting structures | 12.73 | 3 | 2.82 | 3.43 | 9.27E-04 |
| Dental caries | 9.09 | 3.2 | 3.87 | 3.58 | 3.93E-02 |
| Short stature | 3.64 | 21.5 | 0.64 | 5.89 | 1.51E-17 |
| Abnormal weight gain | 7.27 | 5 | 1.38 | 3.04 | 1.16E-08 |
| Lack of normal physiological development | 27.27 | 7.93 | 2.58 | 7.74 | 1.24E-08 |
| Hypokalemia | 9.09 | 7.2 | 1.53 | 5.6 | 4.05E-06 |
| Electrolyte imbalance | 12.73 | 9 | 2.64 | 6.99 | 4.74E-05 |
| Disorders of fluid, electrolyte, and acid-base balance | 16.36 | 8.33 | 6.02 | 6.11 | 1.64E-03 |
| Hypothyroidism | 10.91 | 18.17 | 2.56 | 19.93 | 1.99E-02 |
| Hypothyroidism NOS | 10.91 | 17 | 2.47 | 19.3 | 2.48E-02 |
| Disorders of protein plasma/amino-acid transport and metabolism | 3.64 | 41.5 | 0.45 | 28.64 | 2.50E-02 |
| Retention of urine | 3.64 | 22 | 1.11 | 5.7 | 2.94E-09 |
| Urinary incontinence | 20 | 7.09 | 2.93 | 6.23 | 1.01E-07 |
| Other symptoms/disorders or the urinary system | 30.91 | 9.71 | 14.11 | 5.46 | 1.80E-07 |
| Irregular menstrual cycle | 5.45 | 3.67 | 1.47 | 3.64 | 3.76E-02 |
| Otitis media | 38.18 | 17.33 | 30.87 | 9.5 | 2.89E-04 |
| Otitis media and Eustachian tube disorders | 40 | 17.09 | 32.55 | 9.74 | 7.12E-04 |
| Suppurative and unspecified otitis media | 36.36 | 13.85 | 29.4 | 8.53 | 1.71E-03 |
| Impacted cerumen | 10.91 | 6.83 | 4.85 | 4.4 | 3.51E-03 |
| Other disorders of middle ear and mastoid | 7.27 | 7 | 1.58 | 5.76 | 5.02E-03 |
| Dizziness and giddiness (Light-headedness and vertigo) | 10.91 | 6 | 4.76 | 4.14 | 5.12E-03 |
| Hearing loss | 18.18 | 7.2 | 6.82 | 6.75 | 2.58E-02 |
| Senile cataract | 10.91 | 4.5 | 1.75 | 7.49 | 2.89E-02 |
| Diseases of nail | 20 | 5.45 | 3.69 | 4.22 | 8.51E-11 |
| Pruritus and related conditions | 3.64 | 4 | 1.22 | 2.79 | 1.88E-02 |
| Long-term use of anticoagulants | 5.45 | 76 | 1.02 | 32.46 | 6.71E-07 |
| Complication due to other implant and internal device | 3.64 | 5.5 | 0.55 | 3.1 | 1.26E-06 |
| Other acquired musculoskeletal deformity | 3.64 | 14 | 1.49 | 7.05 | 4.40E-02 |
| Cancer, suspected or other | 3.64 | 30 | 0.35 | 8.79 | 3.60E-12 |
| Pleurisy; pleural effusion | 3.64 | 21 | 1 | 7.55 | 2.74E-05 |
| Influenza | 7.27 | 2.5 | 1.91 | 2.64 | 9.26E-03 |
| Pneumonia | 16.36 | 9.33 | 9.16 | 5.56 | 1.04E-02 |
| Shortness of breath | 5.45 | 16 | 5.36 | 5.63 | 1.31E-02 |
| Rhabdomyolysis | 3.64 | 4 | 0.18 | 5.7 | 5.82E-04 |
| Syncope and collapse | 9.09 | 7.6 | 3.67 | 5.94 | 4.52E-02 |

**Table S3. Physical health conditions associated with FXS in males.** Conditions that were observed in 2 or more cases and survived adjustments for multiple comparisons are listed. Health categories are color coded as follows: circulatory (pink), digestive (green), dental problems (light gray), endocrine/metabolic (orange), genitourinary (white), and other conditions (gray).

|  | **FXS (n=44)** | | **Controls (n=4400)** | |  |
| --- | --- | --- | --- | --- | --- |
| Description | Prevalence  (Percentage) | Average encounters | Prevalence  (Percentage) | Average encounters | P-value  (Encounters) |
| First degree AV block | 4.54 | 4 | 0.16 | 4.14 | 1.61e-07 |
| Dyspepsia and other specified disorders of function of stomach | 6.82 | 7.33 | 0.95 | 3.26 | 1.17e-11 |
| Functional digestive disorders | 20.45 | 7.67 | 5.11 | 4.52 | 8.42e-09 |
| Irritable bowel syndrome | 4.54 | 7.5 | 0.84 | 3.22 | 8.28e-08 |
| Gingival and periodontal diseases | 11.36 | 5.4 | 2.84 | 2.51 | 2.54e-13 |
| Diseases of hard tissues of teeth | 50 | 5.18 | 11.64 | 4.75 | 8.42e-12 |
| Diseases of pulp and periapical tissues | 6.82 | 4 | 1.32 | 2.67 | 7.32e-06 |
| Abnormal weight gain | 9.09 | 5 | 0.89 | 2.87 | 1.19e-16 |
| Short stature | 4.54 | 21.5 | 0.69 | 6.23 | 3.12e-12 |
| Lack of normal physiological development | 27.28 | 8.75 | 2.7 | 7.02 | 3.43e-10 |
| Hypokalemia | 9.09 | 7.75 | 1.23 | 5.02 | 2.99e-08 |
| Electrolyte imbalance | 13.63 | 9.67 | 2.38 | 6.89 | 9.92e-06 |
| Disorders of protein plasma/amino-acid transport and metabolism | 4.54 | 41.5 | 0.37 | 24.38 | 4.08e-05 |
| Urinary incontinence | 20.45 | 7.67 | 2.7 | 5.42 | 3.56e-12 |
| Other symptoms/disorders or the urinary system | 29.55 | 9.77 | 10.27 | 4.91 | 4.12e-11 |
| Retention of urine | 4.54 | 22 | 1.27 | 5.77 | 9.17e-09 |
| Complication due to other implant and internal device | 4.54 | 5.5 | 0.5 | 2.68 | 2.44e-10 |
| Pruritus and related conditions | 4.54 | 4 | 0.57 | 2.76 | 3.73e-06 |

**Table S4. Mental and neurological disorders associated with FXS.** Conditions with significant p-value (<0.05) that were observed in 2 or more cases are listed. Mental disorders are highlighted in purple, neurological disorders highlighted in blue, and congenital anomalies in gray.

|  | **FXS (n=55)** | | **Controls (n=5500)** | |  |
| --- | --- | --- | --- | --- | --- |
| Description | Prevalence  (Percentage) | Average encounters | Prevalence  (Percentage) | Average encounters | P-value  (Encounters) |
| Specific nonpsychotic mental disorders due to brain damage | 34.55 | 12.58 | 1.85 | 8.59 | 2.82E-73 |
| Other specified nonpsychotic and/or transient mental disorders | 38.18 | 12.71 | 3.45 | 7.06 | 4.01E-68 |
| Autism | 25.45 | 113.71 | 1.07 | 39.32 | 6.96E-37 |
| Pervasive developmental disorders | 58.18 | 69.72 | 12.87 | 21.51 | 2.41E-34 |
| Generalized anxiety disorder | 5.45 | 51.67 | 2.09 | 9.52 | 4.78E-32 |
| Psychosis | 7.27 | 14.75 | 0.95 | 6.54 | 3.32E-29 |
| Speech and language disorder | 23.64 | 6.15 | 2.24 | 5.98 | 6.32E-29 |
| Altered mental status | 5.45 | 8.33 | 0.67 | 3.86 | 1.08E-24 |
| Impulse control disorder | 10.91 | 16.5 | 0.91 | 13.02 | 1.60E-16 |
| Attention deficit hyperactivity disorder | 49.09 | 21.85 | 11.53 | 18.8 | 4.84E-16 |
| Developmental delays and disorders | 67.27 | 23.08 | 4.75 | 15.02 | 8.34E-12 |
| Conduct disorders | 45.45 | 15.68 | 5.84 | 15.12 | 1.10E-10 |
| Anxiety, phobic and dissociative disorders | 49.09 | 26.41 | 15.35 | 15.6 | 1.40E-10 |
| Intellectual disability | 49.09 | 25.93 | 0.76 | 48.21 | 4.97E-10 |
| Neurological disorders | 34.55 | 9.79 | 4.42 | 8.74 | 1.70E-09 |
| Schizophrenia and other psychotic disorders | 12.73 | 33.29 | 1.33 | 28.96 | 2.69E-09 |
| Aphasia/speech disturbance | 25.45 | 10.79 | 2.15 | 12.81 | 2.27E-08 |
| Anxiety disorder | 12.73 | 26.43 | 4.98 | 10.62 | 2.51E-08 |
| Other persistent mental disorders due to conditions classified elsewhere | 14.55 | 3.88 | 0.8 | 6.68 | 2.95E-08 |
| Alteration of consciousness | 9.09 | 5 | 1.69 | 4.54 | 4.04E-08 |
| Schizophrenia | 5.45 | 39.67 | 0.6 | 41.91 | 5.99E-06 |
| Posttraumatic stress disorder | 7.27 | 20 | 1.04 | 18.37 | 7.89E-04 |
| Symbolic dysfunction | 3.64 | 23.5 | 0.24 | 20.85 | 2.07E-03 |
| Paranoid disorders | 3.64 | 27.5 | 0.09 | 67.2 | 2.22E-03 |
| Adjustment reaction | 12.73 | 10.57 | 7.55 | 8.33 | 3.61E-03 |
| Psychogenic and somatoform disorders | 3.64 | 4.5 | 0.84 | 4.91 | 1.91E-02 |
| Mood disorders | 41.82 | 19.61 | 14.25 | 19.37 | 2.74E-02 |
| Depression | 18.18 | 18.1 | 11.42 | 14.92 | 4.67E-02 |
| Epilepsy, recurrent seizures, convulsions | 23.64 | 30.85 | 3.07 | 17.91 | 3.15E-15 |
| Convulsions | 18.18 | 12.4 | 2.35 | 8.25 | 3.49E-13 |
| Partial epilepsy | 3.64 | 45 | 0.75 | 17.8 | 6.82E-07 |
| Epilepsy | 3.64 | 55 | 1.24 | 19.54 | 1.99E-05 |
| Insomnia | 3.64 | 16 | 2.55 | 4.69 | 9.34E-04 |
| Abnormality of gait | 7.27 | 13 | 1.55 | 9.02 | 2.11E-03 |

**Table S5.** **Mental and neurological disorders associated FXS in males.** All of the conditions survived adjustments for multiple comparisons. Mental disorders are highlighted in purple, neurological disorders highlighted in blue, and congenital anomalies in gray.

|  | **FXS (n=44)** | | **Controls (n=4400)** | |  |
| --- | --- | --- | --- | --- | --- |
| Description | Prevalence  (Percentage) | Average encounters | Prevalence  (Percentage) | Average encounters | P-value  (Encounters) |
| Pervasive developmental disorders | 68.19 | 71.4 | 13.78 | 21.77 | 3.04e-27 |
| Other specified nonpsychotic and/or transient mental disorders | 34.09 | 9.8 | 3.34 | 6.65 | 8.57e-27 |
| Symbolic dysfunction | 4.54 | 23.5 | 0.25 | 4.18 | 8.81e-27 |
| Specific nonpsychotic mental disorders due to brain damage | 31.82 | 8.93 | 1.8 | 8.47 | 1.31e-24 |
| Autism | 29.55 | 121.69 | 1.14 | 43.54 | 3.61e-20 |
| Neurological disorders | 31.82 | 9.71 | 4.43 | 7.93 | 9.73e-10 |
| Attention deficit hyperactivity disorder | 56.82 | 20.44 | 12.55 | 18.54 | 3.13e-09 |
| Altered mental status | 4.54 | 8.5 | 0.63 | 3.5 | 1.40e-08 |
| Aphasia/speech disturbance | 22.73 | 11.3 | 2.25 | 11.08 | 4.74e-08 |
| Anxiety, phobic and dissociative disorders | 45.45 | 21.35 | 13.37 | 14.09 | 6.90e-08 |
| Conduct disorders | 54.54 | 16.25 | 6.32 | 15.26 | 1.14e-07 |
| Developmental delays and disorders | 72.73 | 19.78 | 4.93 | 14.57 | 2.33e-07 |
| Speech and language disorder | 22.73 | 5.3 | 2.41 | 5.59 | 1.06e-06 |
| Intellectual disability | 56.82 | 20.48 | 0.65 | 56.69 | 1.16e-05 |
| Impulse control disorder | 13.63 | 16.5 | 0.91 | 14.23 | 1.44e-05 |
| Convulsions | 18.18 | 12.75 | 2.48 | 8.06 | 2.23e-09 |
| Epilepsy, recurrent seizures, convulsions | 25 | 19 | 3.2 | 16.08 | 1.94e-07 |
| Insomnia | 4.54 | 16 | 2.18 | 4.22 | 9.99e-07 |
| Chromosomal anomalies and genetic disorders | 100 | 17.61 | 0.43 | 24.32 | 5.30e-228 |

**Table S6. The first twenty conditions differentiating individuals with FXS from controls five years prior to clinical diagnosis, with highest mean decrease in Gini (MDG) coefficient.**

| ICD9 | Description | MDG |
| --- | --- | --- |
| 314.01 | Attention deficit disorder with hyperactivity | 0.0548 |
| 319 | Unspecified intellectual disabilities | 0.033 |
| 599.7 | Hematuria - unspecified | 0.0223 |
| 317 | Mild intellectual disabilities | 0.0197 |
| 382.9 | Unspecified otitis media | 0.0176 |
| 473 | Chronic maxillary sinusitis | 0.0164 |
| 311 | Depressive disorder - not elsewhere classified | 0.0162 |
| V20.2 | Routine infant or child health check | 0.0161 |
| 465.9 | Acute upper respiratory infections of unspecified site | 0.0161 |
| 784 | Headache | 0.0155 |
| 462 | Acute pharyngitis | 0.0147 |
| 312.34 | Intermittent explosive disorder | 0.0147 |
| 295.62 | Schizophrenic disorders - residual type - chronic | 0.0138 |
| 525.9 | Unspecified disorder of the teeth and supporting structures | 0.0128 |
| 783.4 | Lack of normal physiological development – unspecified | 0.0125 |
| 313.81 | Oppositional defiant disorder | 0.0122 |
| 300 | Anxiety state – unspecified | 0.0121 |
| 692.9 | Contact dermatitis and other eczema - unspecified cause | 0.0107 |
| 550.9 | Inguinal hernia - without mention of obstruction or gangrene - unilateral or unspecified (not specified as recurrent) | 0.0106 |
| 780.3 | Convulsions | 0.0103 |

**References**

1. Hunter J, Rivero-Arias O, Angelov A, Kim E, Fotheringham I, Leal J. Epidemiology of fragile X syndrome: A systematic review and meta-analysis. *Am J Med Genet A*. 2014;164(7):1648-1658. doi:10.1002/ajmg.a.36511

2. Breiman L. Random forests. *Mach Learn*. 2001;45(1):5-32.

3. Kingsford C, Salzberg SL. What are decision trees? *Nat Biotechnol*. 2008;26(9):1011-1013.

4. Gajowniczek K, Ząbkowski T, Szupiluk R. Estimating the ROC curve and its significance for classification models’ assesment. *Metody Ilościowe W Badaniach Ekon*. 2014;15(2):382-391.

5. Lever J, Krzywinski M, Altman N. Points of Significance: Classification evaluation. *Nat Methods*. 2016;13(8):603-604.

6. Guyon I, Elisseeff A. An introduction to variable and feature selection. *J Mach Learn Res*. 2003;3:1157-1182.

7. Tang J, Alelyani S, Liu H. Feature selection for classification: A review. *Data Classif Algorithms Appl*. Published online 2014:37.

8. Roobaert D, Karakoulas G, Chawla NV. Information gain, correlation and support vector machines. In: *Feature Extraction*. Springer; 2006:463-470. Accessed January 31, 2016. http://link.springer.com/chapter/10.1007/978-3-540-35488-8_23
